# Teaching the 6 EEG Spectrogram Patterns Using an Infographic

**DOI:** 10.1212/NE9.0000000000200158

**Published:** 2024-09-25

**Authors:** Kaley J. Marcinski Nascimento, M. Brandon Westover, Fábio A. Nascimento

**Affiliations:** From the Department of Neurology (K.J.M.N., F.A.N.), Washington University School of Medicine, St. Louis, MO; and Department of Neurology (M.B.W.), Beth Israel Deaconess Medical Center, Boston, MA.

Quantitative EEG allows faster analysis of continuous long-term EEG recordings—namely in the critical care setting. A standardized, user-friendly nomenclature for EEG spectrograms including 6 patterns has been proposed (Figure).^1^ With high inter-rater agreement among electroencephalographers, this framework serves as a valuable tool to aid in rapid and accurate identification of seizures and ictal-interictal continuum patterns^2^ by experts and nonexperts—including bedside providers. Confirmation on raw EEG should always be pursued. Educators may use Figure and video-based resources^e1^ to teach and illustrate each of the 6 emblematic EEG spectrogram patterns.

**Figure:**
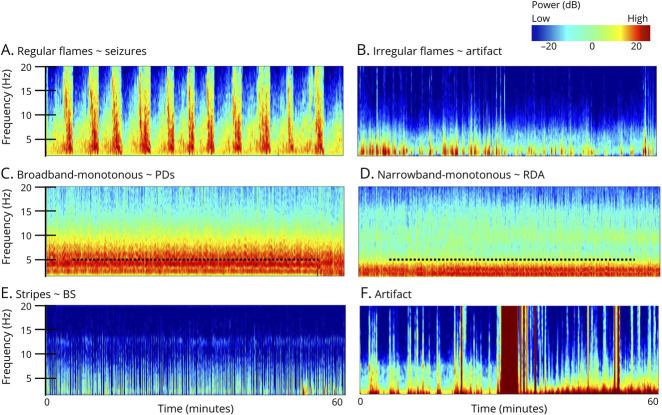
6 EEG Spectrogram Patterns (A) Regular flames ∼ seizures: abrupt segments with high power and bandwidth rising up from delta range. (B) Irregular flames ∼ artifact: choppy and irregular flame-like morphology. (C) Broadband-monotonous ∼ periodic discharges: sustained high power across broad range extending to >5 Hz. (D) Narrowband-monotonous ∼ rhythmic delta activity: sustained high power restricted to <5 Hz. (E) Stripes ∼ burst suppression: alternating low and high power. (F) Artifact: irregular high-power signals across most or all frequencies. ∼ = suggestive of; BS = burst suppression; PDs = periodic discharges; RDA = rhythmic delta activity.

## Appendix Authors

**Table:**

Name	Location	Contribution
Kaley J. Marcinski Nascimento, MD	Department of Neurology, Washington University School of Medicine, St. Louis, MO	Drafting/revision of the manuscript for content, including medical writing for content; major role in the acquisition of data; study concept or design; analysis or interpretation of data
M. Brandon Westover, MD, PhD	Department of Neurology, Beth Israel Deaconess Medical Center, Boston, MA	Drafting/revision of the manuscript for content, including medical writing for content; major role in the acquisition of data; study concept or design; analysis or interpretation of data
Fábio A. Nascimento, MD	Department of Neurology, Washington University School of Medicine, St. Louis, MO	Drafting/revision of the manuscript for content, including medical writing for content; major role in the acquisition of data; study concept or design; analysis or interpretation of data

## References

[1] Hirsch LJ, Fong MWK, Leitinger M, et al. American Clinical Neurophysiology Society's standardized critical care EEG terminology: 2021 version. J Clin Neurophysiol. 2021;38(1):1-29. doi:10.1097/WNP.0000000000000806 33475321 PMC8135051

[2] Zafar SF, Amorim E, Williamsom CA, et al. A standardized nomenclature for spectrogram EEG patterns: inter-rater agreement and correspondence with common intensive care unit EEG patterns. Clin Neurophysiol. 2020;131(9):2298-2306. doi:10.1016/j.clinph.2020.05.032 32660817 PMC7461156

[R3] eReference is available as supplemental digital content at Neurology.org/NE.

